# Sustainable Functional Polymer Composites: Bio-Based Systems with Tailored Properties for Civil Engineering Applications—A Review

**DOI:** 10.3390/polym18101247

**Published:** 2026-05-20

**Authors:** Abdullah Iftikhar, Allan Manalo, Mazhar Peerzada

**Affiliations:** Centre for Future Materials, School of Science, Engineering and Digital Technologies, University of Southern Queensland, Toowoomba, QLD 4350, Australia; allan.manalo@unisq.edu.au (A.M.); mazhar.peerzada@unisq.edu.au (M.P.)

**Keywords:** sustainability, bio-based composites, bio-epoxy: nanomaterials, bio-composites, applications in civil engineering

## Abstract

Conventional epoxy polymers and their composites are increasingly challenged by environmental concerns, high manufacturing costs, and limited recyclability, necessitating the exploration of sustainable alternatives. Many research groups have sought to develop alternate polymers from various renewable resources, such as lignin, polyphenols, natural resins, saccharides, and plant oils. This new type of polymer has led to the emergence of bio-based polymers, which are often used with different reinforcements as bio-based composites. In this review, the synthesis of different bio-epoxy resins is discussed in detail along with their chemical structures. Subsequently, the enhancements in the properties of these bio-composites with the addition of different nanomaterials such as carbonaceous nanofillers (carbon nanotubes, graphene nanoplatelets, graphene oxide, etc.), cellulose-based nanomaterials, inorganic nano-silica (spherical and mesoporous), and nano-clay is explained. Lastly, the properties of these bio-composites and their applications in civil engineering are highlighted. This review has provided a detailed overview of the developments in bio-composites that can be used as a guide for the development of a new class of bio-composites using other alternate resources.

## 1. Introduction

Polymer composites are widely adopted by the civil infrastructure industry because of their excellent strength and durability properties. Petroleum-based epoxy resins are extensively utilised in composite manufacturing. Market analyses indicate a strong and sustained growth trajectory for epoxy thermosets over the past decade. The estimated global market of epoxy thermosets was valued at approximately 7 billion USD in 2015, with projections reaching around 10.2 billion USD by 2021. More recent data suggest a significant expansion, reporting the epoxy resin market at 24.1 billion USD in 2022 and forecasting further growth to approximately 42.28 billion USD by 2030 [1]. However, the problems associated with these resin systems, such as depletion of petroleum-based resources, expensive raw materials for synthesis, and environmental pollution, are becoming a hindrance to the use of conventional epoxy systems [2,3]. These limitations have driven increasing research attention toward the development of sustainable alternatives, particularly renewable bio-based resins, to reduce environmental impact while maintaining the adequate performance essential for civil infrastructure applications.

**Figure 1:**
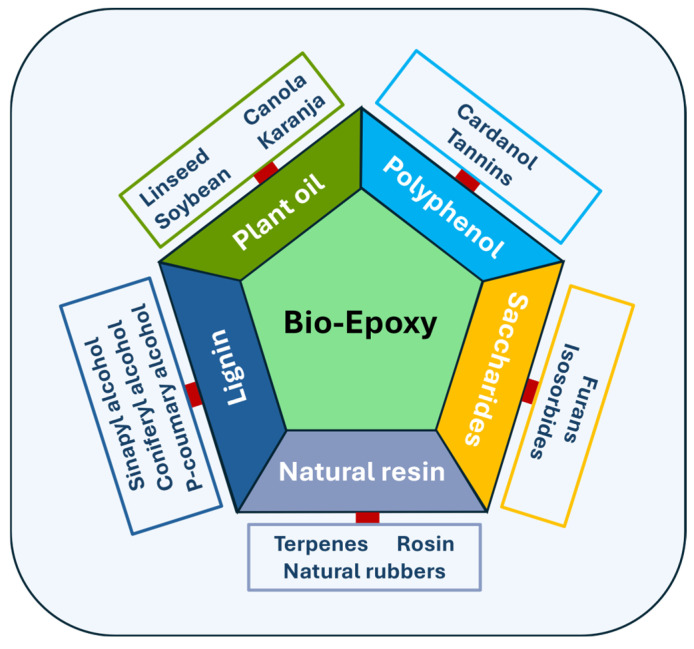
Renewable resources for the synthesis of bio-epoxy resins.

Various research groups have carried out extensive research focused on the development of sustainable alternatives to replace the conventional epoxy resins. The conventional resin systems are mainly synthesised from diglycidyl ether of either novolac or bisphenol-A derived from crude oil [4,5,6], whereas bio-based resin systems are mainly derived from renewable sources [7], including lignin, saccharides, natural resin, polyphenols, and natural oils derived from plants, as shown in Figure 1. The properties of the bio-based resins largely depend on the natural feedstock used for their synthesis. Many researchers focused on the improvement in the characteristic properties of bio-based resin by incorporating nanoparticles such as carbonaceous nanofillers (graphene oxide, graphene nanoplatelets, carbon nanotubes, etc.), cellulose-based nanomaterials, inorganic nano-silica (spherical and mesoporous), and nano-clay. Considering these advancements, a critical analysis of the existing literature is necessary to consolidate current findings and clarify future research directions.

The current review encompasses the synthesis of bio-based resins from renewable resources, highlights the improvements in the intrinsic properties of synthesised resins by the addition of nanoparticles and then assesses the suitability of bio-based nanocomposites in civil engineering applications.

## 2. Synthesis of Bio-Based Resins

### 2.1. Synthesis of Bio-Epoxy from Plant Oils

**Figure 2:**
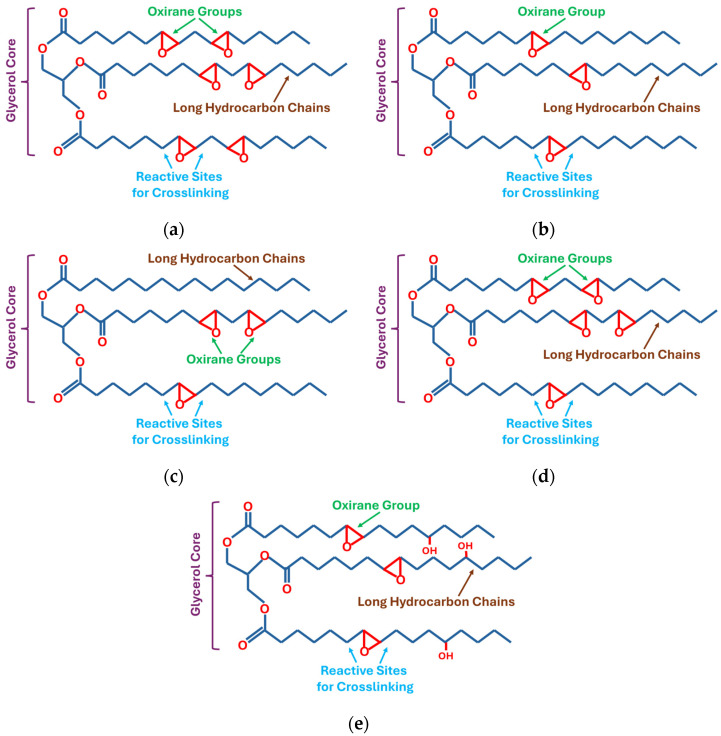
Structures of epoxidised plant oils; (**a**) epoxidised linseed oil, (**b**) epoxidised canola oil, (**c**) epoxidised karanja oil, (**d**) epoxidised soybean oil, (**e**) epoxidised castor oil.

Plant oils containing triglycerides with different molecular structures are one of the renewable resources for the synthesis of bio-epoxy resins. Based on the degree of unsaturation, the plant-based oils are generally divided into three major classes, i.e., drying, semi-drying, and non-drying oils. Linseed oil is the most widely used drying oil, whereas canola and soybean oils are classified as semi-drying oils, and castor and Karanja oils are considered non-drying oils. These oils are used for the synthesis of bio-epoxy resin by the process of epoxidation [8]. Epoxidation is the chemical process of conversion of the carbon–carbon double bond (C=C) into oxiranes. Linseed oil has a long hydrocarbon chain with six double bonds (C=C), which can be converted to oxirane groups after epoxidation. The molecular structure of epoxidised linseed oil with six oxirane groups is shown in Figure 2a. Due to the large number of double bonds available for epoxidation, linseed oil is the preferred renewable source among plant oils for the synthesis of bio-epoxy resin [9]. Likewise, canola oil also has the potential to be epoxidised. It has three carbon–carbon double bonds (C=C) in the hydrocarbon chain, which are converted to three oxirane rings after epoxidation, as shown in Figure 2b. Karanja oil also has three carbon–carbon double bonds (C=C) capable of making three oxirane rings (Figure 2c). Soybean oil has a structure similar to that of linseed oil; however, it typically contains five or six double bonds per triglyceride molecule. The structure of epoxidised soybean oil, with five oxirane groups, is shown in Figure 2d. One of the main advantages of soybean oil is that it is the most economical option among these plant oils [1]. Unlike other vegetable oils, castor oil contains intrinsic hydroxyl groups due to the presence of ricinoleic acid (Figure 2e), which contributes to its reactivity in resin synthesis, in addition to the limited epoxidation of its unsaturated bonds. Overall, the suitability of plant oils for the synthesis of bio-epoxy resins is primarily governed by the number of oxirane groups formed after epoxidation. In this context, linseed oil emerges as the most favourable precursor due to its high degree of unsaturation. However, from an economic and industrial perspective, soybean oil can be considered a suitable alternative, as it provides a comparable oxirane functionality at a comparatively lower cost.

### 2.2. Synthesis of Bio-Epoxy from Saccharides

**Figure 3:**
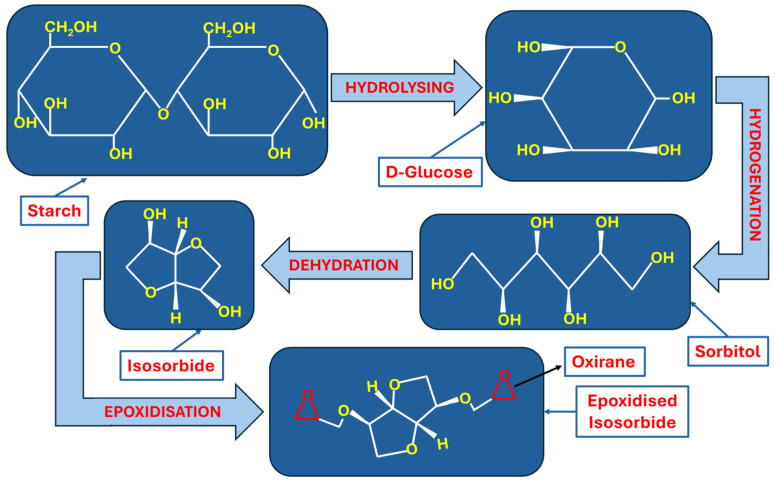
Synthesis of epoxidised isosorbide from starch.

Saccharides are also a renewable source that can be utilised in the fabrication of bio-epoxy resins. Mostly, isosorbides (i.e., polysaccharides) of furans are used for the synthesis of bio-epoxy resins. The synthesis of bio-epoxy resin from starch firstly involves the extraction of isosorbide, as shown in Figure 3. Firstly, the starch was hydrolysed and converted to d-glucose. In the second step, d-glucose was transformed into sorbitol after hydrogenation. Finally, sorbitol was dehydrated to obtain isosorbide, as illustrated in Figure 3. It can be noted the molecular structure of isosorbide is characterised by the presence of two hydroxyl (–OH) groups, which act as reactive sites during epoxidation reactions. These hydroxyl groups, on reaction with epichlorohydrin (ECH), form the final product, i.e., epoxidised isosorbide [4,9].

**Figure 4:**
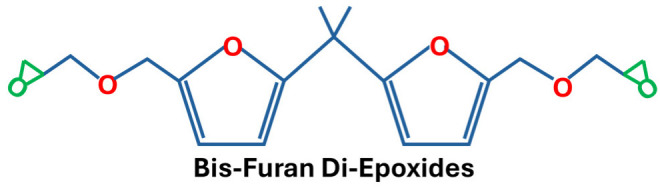

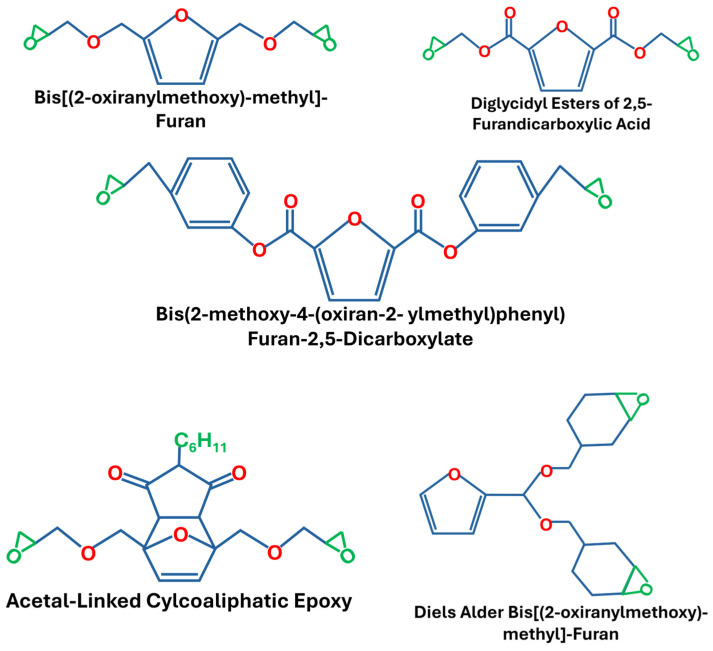
Molecular structures of furans after epoxidation.

Furan-based resins constitute an important class of bio-derived thermosetting polymers obtained from renewable agricultural feedstocks. These compounds are primarily derived from lignocellulosic biomass, where furfural is produced through the catalytic hydrolysis of hemicellulosic sugars. Furfural can subsequently be converted into furfuryl alcohol, which undergoes polymerisation via condensation reactions and Diels–Alder cycloaddition to yield furan-based resin networks. Owing to their heteroaromatic structure and high chemical reactivity, furan derivatives are recognised as key value-added platform chemicals for the development of sustainable polymer systems. In particular, bio-based furan compounds derived from sugar-based resources have attracted considerable attention as precursors for advanced resin formulations. In recent years, significant progress has been made in the synthesis of bio-epoxy resins incorporating furan structures, with various epoxidised furan-based monomers and networks reported in the literature [4,10,11], as shown in Figure 4. These advancements underscore the potential of furan-derived systems as promising alternatives to conventional petroleum-based epoxy resins.

### 2.3. Synthesis of Bio-Epoxy from Polyphenol

**Figure 5:**
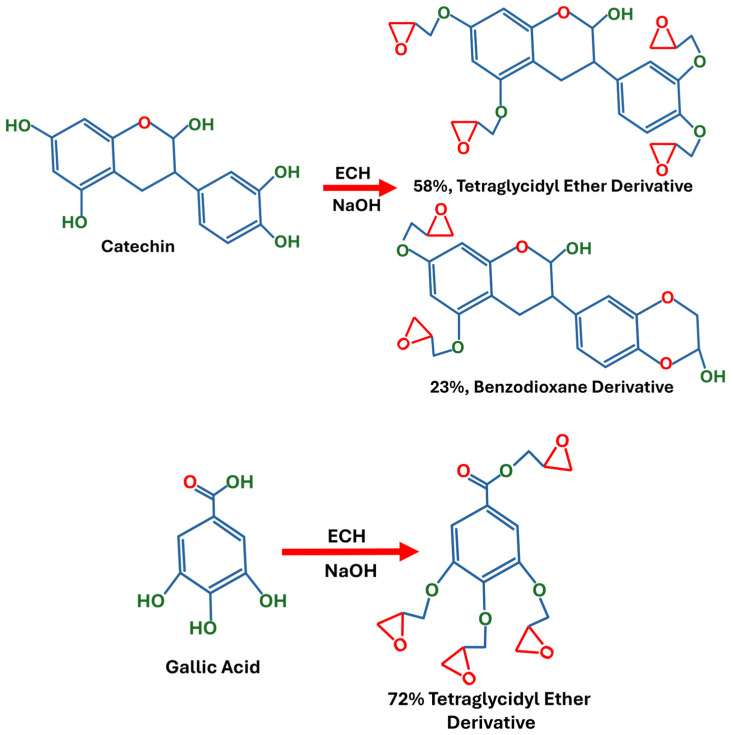
Synthesis of bio-epoxy from catechin and gallic acid.

Cardanol and tannins extracted from various natural sources (i.e., tara, algarobilla chilena, divi-divi, myrabolans, sumach, Uncaria gambir, Acacia catechu, chestnut wood, mangrove wood, quebracho wood, oak bark, and black mimosa bark) are the most suitable polyphenol structures for the synthesis of bio-epoxy resin. The reason for their suitability is their excellent thermal stability and good mechanical properties [12]. Generally, tannins include different compounds such as tannic acid, catechin and gallic acid. Aouf et al. [13] reported that when catechin is treated with epichlorohydrin (ECH) and sodium hydroxide (NaOH), it can be transformed to 58% tetraglycidyl ether derivative and 23% benzodioxane derivative, as shown in Figure 5. They also reported that gallic acid can also be converted to 72% tetraglycidyl ether derivative after reaction with ECH and NaOH, as shown in Figure 5. Kocaman et al. [14] also reported the synthesis of bio-epoxy resin after the reaction of epichlorohydrin (ECH) and fumaric acid.

Tannic acid, belonging to the class of tannins, has been identified as a viable precursor for the synthesis of bio-based epoxy resins and their associated curing agents. Structurally, it is a polyphenolic molecule characterised by the presence of approximately 25 hydroxyl functional groups, which are responsible for its high reactivity. Furthermore, tannic acid is cost-effective, abundantly available worldwide, and its production is largely independent of climatic or seasonal influences [15].

**Figure 6:**
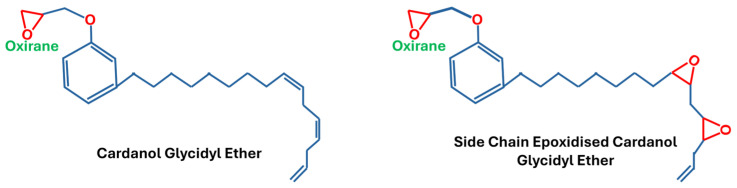

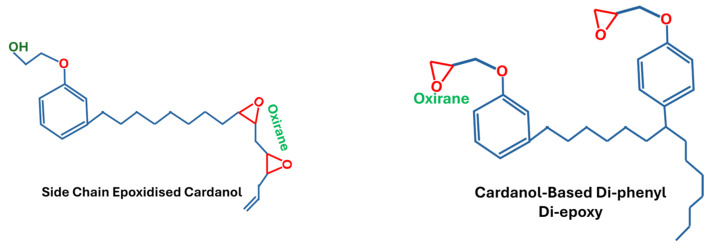
Epoxidised cardanol.

Cardanol is generally obtained from cashew nut shells [16], commonly isolated via vacuum distillation [17]. It is a phenolic lipid featuring a C15 aliphatic side chain. This side chain is composed of a distribution of species, including approximately 5% saturated, 40% mono-unsaturated, 20% di-unsaturated, and 35% tri-unsaturated constituents [18]. As a result, epoxidation of cardanol yields a range of molecular structures, as illustrated in Figure 6.

### 2.4. Synthesis of Bio-Epoxy from Natural Resins

**Figure 7:**
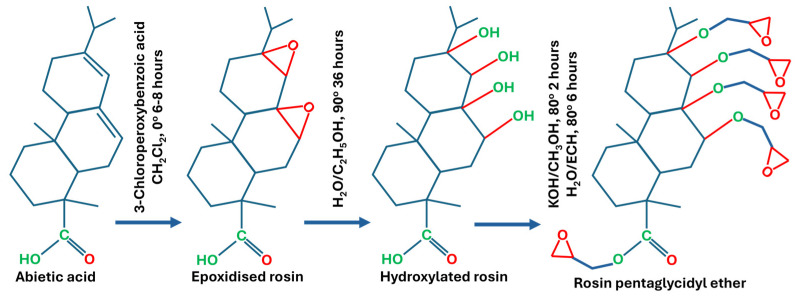
Synthesis of bio-epoxy from abietic acid.

**Figure 8:**
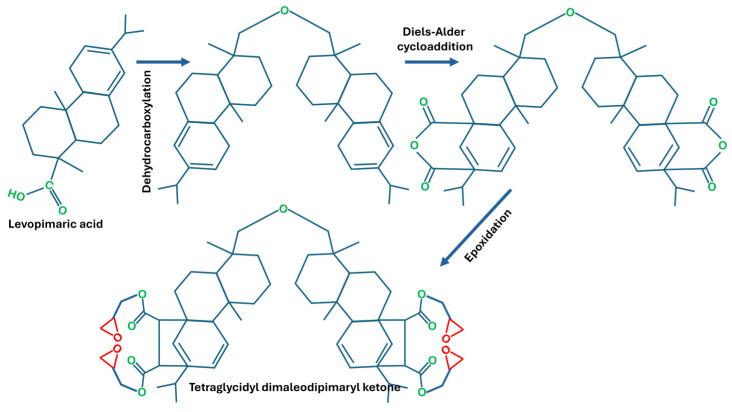
Synthesis of bio-epoxy from levopimaric acid.

Bio-epoxy resins are also synthesised from natural resins, including rosin and natural rubbers extracted from rubber trees. The molecular structure of rosin and rubbers consists of approximately 94% of hydrocarbons with cis-1,4- polyisoprene [19,20]. Resin acids are primarily based on abietane and pimarane-type skeletons, with a general molecular formula of C_19_H_29_COOH. These frameworks exist in multiple isomeric forms, which vary according to their origin (e.g., wood resin, tall oil, or gum) as well as their thermal history. Based on different types of isomers, the epoxy synthesised from rosin also has many distinct structures. Previous studies [21,22] have reported various methods used for the synthesis of bio-based epoxy from rosin acids (i.e., levopimaric and abietic acid), as shown in Figure 7 and Figure 8.

### 2.5. Synthesis of Bio-Epoxy from Lignin

Lignin is widely recognised as the second most abundant natural polymer, following cellulose [23]. In recent years, it has emerged as a particularly attractive renewable source for the development of bio-based epoxy resins, because of its intrinsic aromatic structure and the presence of phenolic hydroxyl groups that can participate in epoxidation reactions [24]. Unlike conventional petroleum-derived epoxy systems, lignin offers a structurally complex and renewable platform for resin synthesis. At the molecular level, lignin consists of a heterogeneous assembly of phenylpropane units, mainly p-hydroxyphenyl, syringyl, and guaiacyl structures. The relative abundance and interlinking of these structural units are highly dependent on the biomass origin and the methods used during processing, resulting in considerable variation in the overall chemical architecture of lignin. Consequently, epoxidised lignin does not exist as a single, well-defined material. Rather, they exist as a family of resins with differing molecular weights and structural configurations. This structural diversity in epoxidised lignin has been explored in several studies. For instance, Zhao et al. [25] synthesised a series of epoxidised lignin resins with varying molecular weights. In a separate study, El Mansouri et al. [26] reported a modified lignin-based epoxy structure while Salanti et al. [27] later proposed an alternative structural model in 2018. Overall, these contributions highlight the adaptability of lignin as a precursor for epoxy resins.

A summary of the employed renewable feedstocks for the synthesis of bio-epoxy resins along with their basic characteristics is listed in Table 1.

## 3. Addition of Nanomaterials for Performance Enhancement

### 3.1. Carbon Nanomaterials

Carbon-based nanofillers have garnered significant research interest over recent decades owing to their diverse morphological forms, including graphene sheets, graphene oxide (GO), carbon nanotubes (CNTs), and carbon nanospheres. This structural diversity enables them to impart a wide range of enhanced functionalities when incorporated into bio-epoxy resin systems. Various researchers have modified the properties of bio-epoxy resin by incorporating different forms of carbon nanomaterials.

The incorporation of graphene oxide (GO) into hyperbranched bio-based epoxy systems has demonstrated notable enhancements in mechanical properties. In the work of Baruch and Karak [36], nanocomposites were synthesised by introducing graphene oxide (GO) in different proportions [0.1–0.5 wt.% (i.e., by weight of resin matrix)] into a hyperbranched bio-epoxy (HBE) matrix synthesised from tannic acid via solution mixing, followed by curing with a poly (amido-amine) hardener. The resulting nanocomposites exhibited considerable enhancements in mechanical performance, with increases of up to 263% in toughness, 159% in elongation at break, 161% in tensile strength, and 109% in adhesion strength, at 0.5 wt.% of graphene oxide. The tensile strength and elongation at break of control HBE were 47 MPa and 24%, respectively, which increased to 76 MPa and 38% after the addition of 0.5 wt.% graphene oxide. Such simultaneous increases in strength and ductility are not typically observed in conventional epoxy systems. This behaviour was attributed to the hyperbranched structure of bio-based epoxy, which provided increased free volume and abundant interaction sites, enabling effective dispersion and interfacial interaction of GO with polymer chains. In addition to mechanical improvements, the study reported enhanced biodegradability, as GO facilitated bacterial proliferation by acting as a scaffold for microorganisms. This combination of improved mechanical performance and bioactivity highlights the potential of GO-reinforced hyperbranched bio-epoxies as sustainable, high-performance materials for advanced engineering applications. Boro and Karak [37] utilised this developed nanocomposite as a protective coating for wood substrates. Their findings indicated that the enhanced adhesion performance was primarily attributed to the synergistic contributions of graphene oxide (GO), the bio-based epoxy matrix, and the poly (amido-amine) curing agent, which together promoted improved interfacial interactions and bonding with the substrate.

Cao et al. [38] studied the use of epoxidised gallic acid, a bio-based polyphenol, to improve graphite/epoxy nanocomposites fabricated using conventional DGEBA epoxy systems. They showed that gallic acid has the capability to get attached to graphene surfaces, which improves the dispersion of graphene in the conventional epoxy matrix. An increment of 47% and 27% was observed in the tensile modulus and tensile strength of graphite/epoxy composites, respectively, with the addition of 2 wt.% of modified graphene. The flexural modulus and strength were also improved by 21% and 9%, respectively. Moreover, thermal and electrical conductivities increased significantly, by around 12 and eight times. These improvements were attributed to the role of epoxidised gallic acid as a bridge between the epoxy and graphene.

Zhao et al. [42] have modified the graphene oxide (GO) by grafting it with bis-furan-di-epoxide (BFDE). Subsequently, they added 0.05–0.5 wt.% of modified GO to synthesise the BFDE nanocomposites. The results indicated that the incorporation of 0.5 wt.% of modified GO resulted in enhancements of 97% in the strain release rate, 80% in tensile strength, 695 in the stress intensity factor, 49% in flexural strength, and 21% in the flexural modulus. Prasomsin et al. [44] utilised epoxidised castor oil as a curing agent for a bio-derived benzoxazine resin. Subsequently, they investigated the impact of carbon nanotubes (CNTs) on their shape memory characteristics. The findings indicated that including 0.1 wt.% by weight of carbon nanotubes (CNTs) yielded optimal performance, achieving shape fixity and recovery ratios of 93% and 98%, respectively. Increasing the concentration of CNTs beyond 0.1 wt.% resulted in a marginal decline in these qualities. A CNT level of 0.5 wt.% markedly lowered the recovery period, reducing it from 36 s to 16 s.

Heydo et al. [28] synthesised a bio-based epoxy from epoxidised linseed oil. The oil was first maleinised and then reacted with diethylenetriamine to form the epoxy network. Reduced graphene oxide (RGO), prepared using a sodium hydroxide treatment, was incorporated into the matrix for coating applications. The results showed a significant improvement in corrosion resistance, with the corrosion rate of mild steel reduced by approximately 5000 times after the addition of RGO. The coating achieved a protection efficiency of 99.98% and remained stable for up to 10 days in a 3.5 wt.% sodium chloride solution, indicating excellent durability in aggressive environments. Madhusudhana et al. [29] have tried to enhance the corrosion resistance of mild steel through a hydrophobic coating system based on PVA and epoxidised linseed oil. In their investigation, amino-functionalised graphene oxide (AGO) was incorporated in epoxidised linseed oil at different proportions, while the PVA to epoxidised linseed oil ratio was kept at 1:1.5. It was found that the nanocomposite incorporated by 0.5% by weight of AGO exhibited the highest charge transfer resistance, indicating superior corrosion protection. This improved performance was attributed to strong crosslinking between the polymer matrix and AGO, which enhanced the coating integrity and limited the penetration of corrosive species. Irfan et al. [30] added graphene oxide (RGO) into a soy-based epoxy system. To further enhance the environmental compatibility and biocompatibility of the coating, sebacic acid was used as a modifier. The resulting nanocomposite exhibited an impedance on the order of 10^6^ Ω·cm^2^, indicating effective corrosion protection performance.

Graphene nanoplatelets (GNPs) are also explored as a useful additive to improve the properties of bio-based epoxy systems. Liu et al. [45] have added GNP in a vanillin-based bio-epoxy and reported substantial enhancement in thermal conductivity of bio-based nanocomposites. It was reported that the thermal conductivity was increased up to ten times after the addition of 10% by weight of GNPs. Owing to these improvements, the developed nanocomposite demonstrates strong potential for thermal management applications, such as heat dissipation components in LED devices. In addition, these bio-based systems offer advantages in terms of sustainability in such a way that the vanillin-based epoxy is degradable. After the degradation of the matrix portion, the GNPs can be recycled easily, making these nanocomposites a sustainable alternative for high-performance thermal conductive applications. Li et al. [46] used jatropha oil, a non-edible feedstock, to synthesise acrylated epoxidised jatropha oil (AEJO) for coating applications. They observed enhanced corrosion resistance by the incorporation of graphene nanoplatelets (GNPs) into AEJO, followed by UV curing. It was also demonstrated that coatings containing 0.5% by weight of GNPs exhibited the most effective protective performance; any further addition caused the agglomeration of GNPs, causing the reduced corrosion resistance.

Hasaan and Bircan [47] epoxidised Tung oil to synthesise bio-based epoxy using amine hardeners (Jeff-amine ED900 and T403). The final bio-epoxy resin was incorporated with 1% by weight of carbon nanofillers, including carbon nanotubes (CNTs), graphene, and fullerene, to formulate coating systems. This work has demonstrated the increased thermal and mechanical performance and enhanced hardness after the addition of nanofillers. However, the adhesion of the formulated coating was reduced with aluminium. In a subsequent study, an alternative curing agent (Jeffamine D2000) was employed to develop similar nanocomposite coatings, reporting comparable enhancements in thermal and mechanical performance [48]. However, the adhesion of the coating layer with the substrate was still not adequate. Later on, Jeffamine D230 was utilised in combination with carbon-based nanofillers to produce coatings for wood substrates [49]. The results demonstrated that the incorporation of these nanofillers significantly improved the resistance to wear and waterproofing of the coatings. These improvements indicated their suitability for protective applications in wood-based materials.

Mahdi et al. [50] used starch for the synthesis of carbon quantum dots (CQDs) using a microwave-assisted pyrolysis method. The synthesised CQDs were then incorporated into a castor oil-based epoxy to produce nanocomposite coatings. The addition of CQDs improved several properties of the system, including the thermal stability, tensile strength, adhesion strength, and viscoelastic behaviour. In addition, the nanocomposite containing 3 wt.% CQDs showed strong capabilities to shield UV by absorbing up to 98% of ultraviolet radiation. Based on these results, the material was proposed for applications such as packaging films for food and pharmaceuticals, as well as protective coatings requiring UV resistance in infrastructure exposed to solar UV radiation.

### 3.2. Nanocellulose

Cellulose nanofibres (CeNFs) are also considered as effective nano-level reinforcements due to their high mechanical performance, light weight, and renewable origin, and they can be incorporated into films, nano-papers, coatings, and bio-epoxy composites to support the development of sustainable materials [51]. Barari et al. [52] modified the bio-epoxy resin by adding 0.9 wt.% and 1.4 wt.% of cellulose nanofibres (CeNFs). The outcome of this work demonstrated that the addition of CeNFs has significantly improved the tribological characteristics of bio-epoxy resin systems. In a separate study, Masoodi et al. [53] also incorporated CeNFs in the bio-epoxy resin and reported considerable improvements in tensile and fracture performance. Wu et al. [54] have utilised cellulose nano-whiskers (CNWs) for the enhancement in the mechanical properties of a new bio-based epoxy system. This new class of bio-epoxy was synthesised from a natural resin, namely, terpene. The CNW dosages incorporated in this bio-epoxy ranged from 0.5 to 8 wt.%. The outcomes revealed a strong reinforcing effect of CNWs in bio-epoxy resin, evidenced by increased modulus and strength. It was reported that the modulus was increased from 295 to 800 MPa, and the tensile strength was doubled compared to the control bio-epoxy resin after the addition of 8 wt.% CNWs.

Although the use of cellulose nanofibres (CeNFs) in petroleum-based epoxy systems has been increasing, the expected improvements in mechanical properties are limited. This is largely because of the weak interaction between the CeNFs and the epoxy matrix, which limits efficient load transfer within the composite. Kuo et al. [55] attempted to overcome this problem by introducing a bio-based epoxy derived from pine bark to the petroleum-based epoxy. The bio-resin was formulated by the reaction with epichlorohydrin, and its composition was largely catechin-dominant. The presence of carbon nanofibres (CNFs) resulted in a pronounced reinforcing effect. The modulus and tensile strength increased to about 298% and 88%, respectively. Also, adding 10 wt.% of bio-epoxy led to an increment in toughness of about 84%. Subootina et al. [56] added carbon nanofibres (CNFs) into a specifically synthesised bio-based epoxy system, emphasising the recyclability. The epoxy resin, produced from vanillin, was incorporated with labile acetal and ester bonds to facilitate controlled degradation. This design enabled the matrix to be selectively depolymerised into its original monomeric components, with the recovery percentages reported between 63% and 95%. The suggested fabrication and recycling method present a feasible strategy for creating thermoset composites with closed-loop recyclability. This can significantly lower the environmental impact of polymer composite waste.

Yue et al. [57] synthesised a phenolic-based bio-based resin from n-alkanols and di-phenolic acid (DPA) obtained from sugar fermentation (e.g., ethanol and methanol). DPA possesses a chemical structure analogous to bisphenol-A, as it is synthesised from levulinic acid [58]. After the production of the epoxy, cellulose nanocrystals (CNCs) were added into the system. The surface modification was carried out with an amine-functional silane to enhance their interaction with the epoxy matrix. As a result, the mechanical properties were improved significantly. The storage modulus was increased from 19.5 MPa for the unmodified resin to 151.1 MPa for the nanocomposite with 10 by weight of modified CNCs at 160 °C. Aziz et al. [59] synthesised cellulose nanocrystals (CNCs) from cotton and subsequently changed their surface with a eugenol-based epoxy silane. The tailored CNCs were then integrated into the SIEEP4 and SIEEP2 bio-epoxy systems, which originate from the hydrosilylation of epoxidised eugenol. The addition of modified nanofillers increased the interfacial adhesion within the epoxy matrix. An increment in coating toughness was observed, which demonstrates the beneficial effects of surface-modified CNCs on the properties of bio-based epoxy coatings.

### 3.3. Nano-Clay

Nano-clay particles are regarded as efficient reinforcements for epoxy systems. Sodium montmorillonite is one of the most prevalent varieties, comprising layered silica and alumina structures [60]. This stratified morphology significantly influences performance. The layers can stop the crack growth under mechanical loading by redirecting the cracks. In corrosive environments, they serve as barriers that stop the ingress of corrosive species into the epoxy matrix. Because of these attributes, nano-clay has been progressively investigated as a reinforcing component in bio-epoxy systems. Several investigations are concentrated on enhancing mechanical durability and corrosion resistance by using nano-clay particles.

Cedeno and Schmidt [39] examined the curing behaviour of a bio-epoxy system after adding 5 wt.% of cloisite 93A nano-clay. The epoxy matrix utilised sorbitol glycidyl ether (SGE) with various curing agents including triethylenetetramine (TETA), polypropylene oxide (PPO), and polyethylene oxide (PEO). The maximum degree of polymerisation (96%) was noted in the SGE–PEO system using nano-clay. This formulation demonstrated higher energy absorption, with an absorbed energy of 0.8 J. On the other hand, the nanocomposites treated with TETA had improved mechanical properties, achieving a hardness of 80.6, an elastic modulus of 1.7 GPa, and a tensile strength of 40.5 MPa. Cedeno et al. [61] investigated a bio-epoxy reinforced with nano-clay by combining sorbitol glycidyl ether (SGE) with a castor oil-based epoxy in different proportions (30:70 to 90:10). The formulations were cured with a dimer diamine, while cloisite 20B and cloisite 30A served as nano-clay fillers. The optimal curing degree was attained with the formulation comprising 70% castor oil epoxy AND 30% SGE, achieving approximately 98% and 99% for the cloisite 20B and cloisite 30A system, respectively. The nanocomposites with cloisite 30B consistently exhibited superior mechanical behaviour compared to those with cloisite 20A across all blending ratios. Kodali et al. [62] incorporated nanoparticles of clay sourced from Georgia and Brazil into a Super Sap Entropy bio-epoxy system. The particles were treated with decalin and ammonium salt to improve their compatibility with the resin matrix before addition. The use of these modified clays resulted in a significant enhancement in flexural characteristics. The strength and modulus increased by around 23–38% and 28–37%, respectively. The improvements in thermal stability can be evidenced by an elevation of around 7–25 °C in the primary degradation temperatures from the TGA study. Moreover, DMA and TMA data indicated a 6–64% enhancement in the coefficient of thermal expansion (and a slight rise in storage modulus (4–6%)), substantiating the reinforcing influence of the nano-clay in the bio-epoxy system.

An increasing trend in using bio-epoxy systems synthesised from resins generated from vegetable oils reinforced with nano-clay has been seen in recent years. Soybean and castor oil are the most common precursors for synthesising bio-epoxy. This is due to their accessibility and appropriate chemical structure for epoxidation. Palavai et al. [31] initially synthesised acrylated epoxidised castor oil (AECO) and employed it as a toughening agent in combinations with DGEBA epoxy. The research examined the impact of montmorillonite (MMT) on the healing behaviour of these systems. The findings indicated that the use of 1 wt.% nano-clay in blends comprising 10 wt.% and 20 wt.% AECO resulted in favourable curing properties. The authors proposed that these formulations may act as viable replacements to traditional DGEBA-based epoxy systems for industrial applications. In a later work [32], they assessed the mechanical characteristics of a DGEBA with 1 wt.% montmorillonite with 20 wt.% AECO blends fortified. The nanocomposite demonstrated a modulus of 1.7 GPa and tensile strength of 50 MPa. The flexural strength and modulus were measured as 120 MPa and 3.02 GPa, respectively. The elongation at break was documented as 19%. The fracture energy and toughness were 1.8 kJ/m^2^ and 2.5 MPa·m^0.5^, respectively. Thermal analysis indicated enhancements relative to the unfilled system, exhibiting a char yield of 8.9% at 700 °C and an elevated glass transition temperature of 108 °C [33]. Saikia and Karak [34] synthesised linear and hyperbranched bio-epoxy resins from castor oils and evaluated the effect of 1–3 wt.% montmorillonite addition. The linear epoxy was synthesised utilising calcium oxide, glycerol, and castor oil as a catalyst. Meanwhile, the hyperbranched system was produced through reactions of epichlorohydrin, triethanolamine, monoglyceride, and bisphenol A derived from castor oil. The integration of montmorillonite results in significant performance enhancements. Both the tensile strength and scratch resistance were doubled, and thermal stability improved from 213 to 316 °C. The hyperbranched epoxy nanocomposites showed better properties compared to the linear bio-epoxy in mechanical, thermal, and chemical performance.

### 3.4. Nano-Silica

Nanoparticles of silica are considered the most economical and adaptable fillers for mesoporous or solid polymer structures [63,64]. These are commonly incorporated in epoxy resins for the improvement in various attributes like corrosion resistance and wear resistance in coatings. Therefore, the incorporation of these nanoparticles into bio-epoxy systems is expected to play an increasingly important role in the development of sustainable nanocomposites. Consequently, the incorporation of these nanoparticles into bio-epoxy resin as green nanocomposites is likely to increase in future.

Zheng et al. [40], in 2020, integrated two varieties of nano-silica into an isosorbide-based bio-epoxy, comprising mesoporous silica (~200 nm) and spherical silica particles (5–15 nm). The findings demonstrated that a loading of 25 wt.% silica yielded optimal performance, achieving a corrosion inhibition efficiency of around 93.75% in hostile settings. The coating demonstrated pronounced hydrophobic properties, evidenced by a water contact angle of 153.0° and a sliding angle of 14.3°. In a subsequent investigation [41], the same group further established that this nanocomposite exhibited excellent mechanical endurance under sand erosion conditions (10–30 μm particles), affirming its appropriateness for protective coating applications.

Aziz et al. [65,66] formulated a silicon-containing bio-epoxy utilising a silicone-derived precursor, resulting in a silicon-bridged difunctional epoxy architecture. They subsequently integrated silsesquioxane nanoparticles at loadings of 1, 3, and 5 wt% to examine their influence on mechanical performance. The findings indicated that the nanocomposite containing 3 wt.% nanoparticles demonstrated the highest tensile modulus, but the peak tensile strength was attained at 5 wt.%. The enhancements were associated with the crystallisation behaviour of the nanoparticles, which facilitated superior reinforcing within the epoxy matrix.

Bifulco et al. [43] first synthesised a bio-based epoxy system by coupling aminopropyl triethoxysilane (APTS) with 2,5-bis[(oxiran-2-ylmethoxy) methyl]furan (BOMF). After that, silica was synthesised with tetraethylorthosilicate (TEOS) and incorporated into the resin. This was followed by curing with methyl nadic anhydride (MNA). A comparable system loaded with silica nanoparticles was also formulated using a traditional petroleum-based epoxy. The findings indicated that both systems demonstrated similar morphological and thermal characteristics, with only negligible discrepancies noted. This indicates that the bio-based nanocomposite can efficiently substitute its petroleum-based equivalent without sacrificing performance.

Li et al. [67] synthesised a novel bio-based epoxy, 2,2′-diglycidyl ether-3,3′-dimethoxy-5,5′-diallyl diphenylmethane (BEF-EP), and employed it to fabricate nanocomposites including diatomite as a porous silica-rich filler. The incorporation of 20 wt% diatomite resulted in significant enhancements in the thermal stability, hydrophobic properties, and storage modulus of the epoxy system. The observed improvements were attributed to the porous structure and elevated SiO_2_ concentration of diatomite, which improve reinforcing and barrier qualities within the matrix. Ji et al. [68] synthesised a bio-based elastomer named as poly(dimethyl itaconate-co-butadiene) (PDMIB). The synthesis involved copolymerisation of butadiene and dimethylitaconate under moderate circumstances. The elastomer was then epoxidised and utilised to fabricate nanocomposites by adding silica nanoparticles. The resultant silica/bio-elastomer system demonstrated potential for several practical applications where flexibility and superior mechanical performance are essential. The typical examples include bio-based gloves, high-performance tyres, and conveyor belts.

### 3.5. Other Nanomaterials

In addition to the nanofillers discussed above, many other nanomaterials are available; however, their application in bio-epoxy resins is not well explored, for example, nano-TiO_2_, nano-zirconia, chitin nanofibres, etc. Aung et al. [69] investigated the effects of the addition of zinc oxide nanoparticles (ZnO) into acrylated epoxidised jatropha oil for corrosion protection. They added several proportions ranging from 0 to 9 wt.%. The findings indicated that 5 wt.% of ZnO offers the optimal corrosion protection. Saikia et al. [70] examined the corrosion resistance of bio-epoxy coatings by adding polyaniline fibres (PAFs) through an in situ approach. The epoxy matrix was synthesised via the polycondensation of bisphenol A, sorbitol, and castor oil. The incorporation of PAFs enhanced the protective efficacy of the coating. The nanocomposite exhibited a corrosion rate of 5.68 × 10^−3^ miles per year in a 3.5% NaCl solution, demonstrating superior resistance compared to the unmodified epoxy system.

Shibata et al. [71] formulated nanocomposites with sorbitol polyglycidyl ether reinforced with chitin nanofibres. This study notably utilised chitosan as a curing agent, contrasting it with traditional hardeners like poly(amidoamine) (PAA) and polyether amine (PEA). An increase in nanofibre content resulted in a minor decrease in transparency for both chitosan- and PAA-cured systems. The chitosan-cured nanocomposites exhibited superior mechanical characteristics. At 3 wt.% nanofibre loading, the modulus and tensile strength attained values of 2390 MPa and 49.6 MPa, respectively. A related study [71] investigated the use of amino acids, including lysine, arginine, and cysteine, as curing agents for bio-epoxy systems derived from chitin and chitosan. The findings demonstrated that the ideal nanofibre concentration was approximately 10 wt.% for chitin and 2 wt.% for chitosan when utilising these amino acid hardeners.

Yang et al. [72] utilised lignin derived from moso bamboo as a sustainable precursor to develop a lignin-based epoxy system. Lignin was initially epoxidised and subsequently integrated with titanium dioxide nanoparticles to create hybrid nanoparticles. These particles were then added to the epoxy matrix to improve its performance. The findings indicated that the incorporation of 10 wt.% of these nanoparticles enhanced antibacterial activity, UV protection, thermal stability, and mechanical performance.

Wang et al. [73] synthesised nanocomposites utilising a bio-derived bisphenol A epoxy (Dow Plastics) incorporated with chitin nano-whiskers. The incorporation of nano-whiskers resulted in a significant improvement in the thermal and mechanical properties. It was reported that at a 2.5 wt.% addition, the elongation at break, toughness, modulus, and tensile strength jumped by roughly 250%, 457%, 16%, and 49%, respectively. The improvements occurred mostly due to the presence of amine groups on the nano-whiskers. These nano-whiskers facilitate excellent interfacial bonding with the epoxy matrix, leading to efficient stress transfer within the composite.

A summary of the nano materials added in bio-epoxy and the consequent enhancements in the properties are presented in Table 2.

## 4. Applications

This research highlights the significant potential of bio-based epoxy systems for civil engineering applications, particularly in areas where traditional epoxy resins are used as coatings, adhesives, matrices for fibre-reinforced composites, and protective surface layers. Their significance to the construction industry stems not only from their renewability but also from demonstrated enhancements in corrosion resistance, adhesion, toughness, hydrophobicity, abrasion resistance, and thermo-mechanical stability in several of these materials. The performance characteristics are directly linked to the functional specifications of infrastructure materials subjected to severe service conditions.

### 4.1. Protective Coatings for Steel and Concrete

One of the most immediate application areas is in protective coatings for steel and reinforced concrete infrastructure. Several studies reviewed in this manuscript have shown that bio-epoxy systems derived from linseed oil, soybean oil, isosorbide, jatropha oil, and castor oil can provide excellent barrier performance against aggressive species. For instance, reduced graphene oxide-modified epoxidised linseed oil coatings substantially reduced the corrosion rate of mild steel and achieved very high protection efficiency in saline environments [28]. Likewise, amino-functionalised graphene oxide in linseed oil-based systems [29] and reduced graphene oxide in soy-based epoxy [30] improved coating integrity and electrochemical resistance by restricting the penetration of corrosive media. Similar benefits were observed for nano-silica and ZnO-modified bio-epoxy coatings, which exhibited high corrosion inhibition and improved resistance to erosive action [40,69]. These findings indicate that bio-based epoxy coatings are especially relevant for steel components in bridges, marine and coastal infrastructure, pipelines, storage tanks, and industrial facilities, where chloride-induced corrosion is a dominant deterioration mechanism.

### 4.2. Strengthening and Repair Materials

A second promising domain is the application of bio-based epoxy systems as a matrix and adhesive components in reinforcement and repair materials. In civil engineering, epoxy resins are extensively utilised in externally bonded fibre-reinforced polymer (FRP) systems, crack injection, structural bonding, and repair mortars. For these applications, sufficient tensile strength, fracture resistance, adhesion, and environmental stability are needed. The reviewed research indicates that graphene oxide, nanocellulose, nano-clay, chitin nanostructures, and silica-based fillers can significantly enhance the mechanical properties of bio-epoxy matrices. The concurrent enhancement in strength, elongation, and toughness shown in tannic acid-based hyperbranched epoxy/graphene oxide systems is appealing for bonded repair materials, where the prevention of brittle fracture is essential. Likewise, cellulose-based nanofillers [52,53,54] and chitin nano-whiskers [73] enhanced the tensile strength, modulus, and toughness, suggesting their potential application in bio-based adhesives and composite matrices necessitating effective stress transfer. While numerous systems remain unvalidated in extensive strengthening applications, their property profiles indicate potential as more sustainable alternatives to petroleum-based binders in non-critical or semi-structural repair applications, and, pending additional durability validation, possibly in structural strengthening systems.

### 4.3. Surface Protection and Wear-Resistant Applications

Bio-epoxy nanocomposites demonstrate potential in surface protection and wear-resistant applications pertinent to civil infrastructure. Coatings for flooring, timber components, façade elements, protective panels, and transportation infrastructure necessitate adhesion and environmental resistance, as well as durability against wear, scratching, and recurrent surface damage. In this context, nanocellulose-reinforced systems showed superior tribological performance [52] whereas nano-clay and silica-modified systems exhibited increased hardness, scratch resistance, and erosion resistance [39,41,61,62]. Nanocomposite coatings derived from plant oils, formulated for wood substrates, exhibited enhanced wear resistance and water repellence [49], indicating potential applications in timber buildings, engineered wood panels, and architectural components subjected to moisture. These applications may facilitate the initial adoption of bio-based epoxy systems in the field, as the performance requirements are typically less stringent than those for core structural components, but the sustainability advantages remain considerable.

### 4.4. Multifunctional Materials for Infrastructure Systems

Another emerging direction is the development of multifunctional materials for infrastructure systems. Unlike conventional binders that provide only mechanical bonding, several of the reviewed bio-epoxy systems exhibit additional functionalities such as UV shielding, hydrophobicity, thermal conductivity, antibacterial performance, and shape memory behaviour. For example, carbon quantum dot-modified castor oil epoxy showed strong UV-blocking capacity [50], which may be beneficial for outdoor coatings exposed to intense solar radiation. A lignin-based epoxy containing TiO_2_ nanoparticles displayed improved UV resistance and antibacterial activity [72], offering potential for hygienic and weather-resistant surfaces in public infrastructure. In addition, thermally conductive graphene-based bio-epoxy systems demonstrated significant improvements in heat dissipation [45], suggesting potential for niche infrastructure applications involving embedded electronics or smart monitoring systems. While such applications remain at an early stage, they align with the broader transition toward multifunctional and smart infrastructure materials.

Despite these encouraging developments, the translation of bio-based epoxy systems into civil engineering practice still requires careful consideration. Most studies reported in the literature focus on short-term mechanical, thermal and electrochemical performance under laboratory conditions, whereas civil materials must maintain reliable performance over long service periods under moisture exposure, alkaline environments, saline conditions, temperature fluctuations, ultraviolet radiation and sustained loading. Therefore, the real suitability of these systems for infrastructure applications depends not only on their initial performance but also on their long-term durability, interfacial stability and resistance to environmental degradation. This is particularly critical for applications involving FRP composites and bonded interfaces, where the resin plays a key role in load transfer and damage evolution. Accordingly, future development of bio-based epoxy systems for civil engineering should prioritise durability-driven assessment under realistic service environments.

## 5. Durability Considerations for Civil Engineering Applications

A limited number of studies have investigated the long-term durability of bio-based epoxy systems under environmental conditions relevant to civil engineering applications. Iftikhar et al. [5] evaluated glass fibre single-yarn composites manufactured using bio-epoxy, epoxy, and vinyl ester resins under hygrothermal exposure at 60 °C and 98% relative humidity for up to 3000 h. The study showed that bio-epoxy exhibited comparatively stable thermal and chemical behaviour, with only a 10% reduction in tensile strength and 6% reduction in interfacial shear strength, whereas conventional epoxy and vinyl ester systems exhibited more pronounced hydrolytic degradation and interfacial deterioration. Similarly, Iftikhar et al. [7] investigated bio-epoxy composites reinforced with carbon, glass, basalt, and flax fibres and reported that the degradation mechanisms strongly depended on fibre type. Carbon fibre composites exhibited the highest durability with only a 4% reduction in interfacial shear strength, while flax fibre composites showed greater interfacial degradation because of moisture-induced fibre swelling and fibre pull-out. Senselova et al. [3] further examined glass- and carbon fibre composites fabricated using epoxy, vinyl ester, and bio-epoxy matrices under hygrothermal ageing at temperatures up to 60 °C for 125 days. Their results indicated that carbon fibre composites demonstrated higher environmental stability than glass fibre systems, while bio-epoxy matrices exhibited greater susceptibility to moisture absorption and interlaminar shear strength degradation despite maintaining relatively stable tensile properties. In addition, Lopez-Arraiza et al. [74] investigated seawater immersion effects on basalt/flax hybrid bio-epoxy composites and reported reductions in the tensile, flexural, and impact properties after prolonged immersion because of moisture ingress and fibre–matrix interfacial degradation. Olatunbosun et al. [75] also observed progressive mechanical degradation in alkali-treated sunn hemp fibre/bio-epoxy composites during hygrothermal ageing, primarily associated with moisture absorption, swelling, and weakening of the fibre–matrix interface. Although these studies demonstrate that certain bio-based epoxy systems can retain acceptable performance under accelerated environmental ageing, the available literature remains very limited and is largely restricted to short-term laboratory exposure under isolated environmental conditions. Comprehensive investigations involving alkaline environments, saline exposure, freeze–thaw cycling, combined environmental actions, ultraviolet weathering, and sustained mechanical loading are still largely absent.

## 6. Cost Comparison

Table 3 presents a cost comparison between conventional DGEBA and several representative bio-based epoxy feedstocks. The reported values were estimated from the previous literature [12,76,77,78,79,80,81,82] and current industrial market trends. Therefore, they should be considered approximate comparative indicators rather than exact economic values, as the actual cost may vary depending on feedstock purity, geographic region, processing scale, energy price, and manufacturing approach. Among the renewable precursors, soybean oil appears to be the most economically attractive option because of its low raw material cost, relatively low epoxidation energy demand, and well-established industrial processing infrastructure. Linseed oil exhibits slightly higher processing and feedstock costs; however, its greater degree of unsaturation enables higher oxirane functionality, making it technically favourable for epoxy synthesis. In contrast, lignin- and isosorbide-based systems exhibit substantially higher overall production costs despite their renewable origin. This increase is primarily associated with multi-step purification, depolymerisation, glycidylation, and solvent-intensive processing requirements, which significantly increase catalyst consumption, energy demand, and downstream separation costs. Although DGEBA remains economically competitive due to highly optimised petrochemical manufacturing and mature industrial infrastructure, bio-based systems continue to gain interest because of their lower environmental impact, low cost of several renewable feedstocks, and renewable origin. Their production cost is expected to decrease further through process optimisation and large-scale industrial implementation.

## 7. Future Research

Despite significant progress in the development of bio-based epoxy systems, several challenges must be addressed before their widespread adoption in civil engineering applications. One important direction is the development of fully bio-based systems, including resins, curing agents, and nanofillers derived entirely from renewable sources. Although many bio-epoxy matrices have been successfully synthesised from lignin, plant oils, saccharides, and polyphenols, the reliance on petroleum-based hardeners remains a limitation. Future research should emphasise the application-specific design of bio-epoxy systems, where material properties are tailored according to infrastructure requirements. For example, enhanced adhesion and toughness are essential for repair and strengthening systems, while improved barrier properties and hydrophobicity are critical for protective coatings exposed to aggressive environments. A major research gap lies in the limited understanding of long-term durability under realistic service conditions. Bio-epoxy systems must be evaluated under hygrothermal ageing, alkaline and saline exposure, UV radiation, and cyclic temperature variations to assess degradation mechanisms such as plasticisation, hydrolysis, and interfacial debonding. In addition, the interfacial behaviour between bio-epoxy matrices and fibres or substrates requires further investigation, particularly for applications involving fibre-reinforced polymer systems and bonded joints. Finally, the development of recyclable thermoset networks and the validation of these materials through large-scale and field-based studies will be essential for enabling their practical implementation in sustainable civil infrastructure.

## 8. Conclusions

In this review, recent advances in sustainable bio-based epoxy systems and their nanocomposites were critically examined with emphasis on their relevance to civil engineering applications. Various renewable feedstocks, including plant oils, saccharides, lignin, polyphenols, natural resins, and natural rubber, were first discussed as precursors for the synthesis of bio-epoxy resins, together with the influence of their molecular structures on network formation and final material performance. Subsequently, different categories of nanomaterials, including carbonaceous nanofillers, nano-clay, nanocellulose, nano-silica, chitin nanostructures, and metal oxide nanoparticles, were evaluated as reinforcing phases for improving the thermo-mechanical, tribological, anticorrosive, hydrophobic, UV-shielding, and multifunctional characteristics of bio-epoxy systems. The reviewed studies demonstrated that the incorporation of nanofillers can significantly enhance interfacial interactions, crack resistance, barrier performance, and load transfer efficiency, thereby expanding the applicability of bio-epoxy nanocomposites in protective coatings, strengthening systems, repair materials, and multifunctional infrastructure components. In addition, several investigations showed that bio-derived epoxy structures can also be incorporated into conventional petroleum-based epoxy matrices to improve sustainability while maintaining desirable engineering performance. The development of recyclable thermoset networks, bio-based curing agents, multifunctional hybrid nanofillers, and durability-oriented bio-epoxy formulations represents an emerging direction in this field. Nevertheless, the current literature remains largely dominated by short-term laboratory evaluations, while long-term durability under realistic environmental conditions relevant to civil infrastructure is still insufficiently understood. Consequently, future research should prioritise degradation mechanism-based durability studies, scalable processing strategies, and field-oriented validation to facilitate the practical implementation of sustainable bio-epoxy nanocomposites in structural and infrastructure applications.

## Figures and Tables

**Table 1:** Summary of renewable feedstocks used for synthesis of bio-based epoxy systems.

Feedstock	Bio-Epoxy System	Properties	Advantages	Limitations	References
Linseed oil	Epoxidised linseed oil	ELO-based matrices showed substantial corrosion protection after rGO/AGO modification (68 °C < *T_g_* 134 °C)	High oxirane functionality due to high unsaturation; favourable for crosslinked coating networks	Aliphatic backbones may limit stiffness, *T_g_*, and thermal resistance relative to aromatic epoxies	[8,9,28,29]
Soybean oil	Epoxidised soybean oil (ESO)/soy-based epoxy	Soy-based epoxy/rGO coating exhibited impedance of ~10^6^ Ω·cm^2^, indicating effective anticorrosion performance	Economical, commercially available, and scalable renewable feedstock	Lower intrinsic stiffness and thermal resistance than rigid aromatic bio-epoxies	[1,8,30]
Castor oil	Epoxidised or acrylated epoxidised castor oil (AECO); castor oil-based hyperbranched epoxy	AECO/DGEBA/MMT system reported tensile strength of 50 MPa, modulus of 1.7 GPa, flexural strength of 120 MPa, flexural modulus of 3.02 GPa, elongation at break of 19%, fracture energy of 1.8 kJ/m^2^, fracture toughness of 2.5 MPa·m^0.5^, and *T_g_* of 108 °C	Hydroxyl functionality supports network formation; improves toughness and flexibility	High aliphatic content can reduce rigidity and heat resistance	[31,32,33,34]
Canola oil	Epoxidised canola oil	13 min < Gel time at 155 °C < 28 min. −3.6 °C < *T_g_* < 37.8 °C	Renewable plant oil source with moderate oxirane functionality	Fewer oxirane groups than linseed oil; limited performance data in the reviewed manuscript	[8]
Karanja oil	Epoxidised Karanja oil	109 °C < *T_g_* 112.7 °C and tensile strength range from 4.5 MPa to 10.6 MPa	Non-edible renewable feedstock	Limited optimisation and property data	[8]
Cardanol	Epoxidised cardanol derivatives	Based on E’ peaks *T_g_* ranges from 15 °C to 50 °C. Based on DSC *T_g_* ranges from 37 to 60 °C. 7 MPa < *E′* at 20 °C < 1218 MPa	Hydrophobicity, flexibility, and renewable phenolic structure	Long aliphatic side chain may reduce modulus and *T_g_*	[16,17,18]
Tannic acid	Epoxidised tannic acid/tannic acid-based hyperbranched epoxy	Neat hyperbranched bio-epoxy reported tensile strength of 47 MPa and elongation at break of 24%; after 0.5 wt.% GO, tensile strength increased to 76 MPa and elongation to 38%	High functionality; aromatic structure; strong interaction sites for nanofillers	Complex curing behaviour and possible brittleness if network density is excessive	[15,35,36,37]
Catechin/gallic acid	Glycidyl ether derivatives of catechin and gallic acid	*T_g_* of 178 °C based on DSC thermograms	Aromatic bio-based structure; improves compatibility with graphitic nanofillers	Multi-step synthesis; limited neat-resin mechanical data in the manuscript	[13,38]
Isosorbide	Epoxidised isosorbide/sorbitol glycidyl ether-related systems	SGE–TETA/nano-clay system reported hardness of 80.6, elastic modulus of 1.7 GPa, and tensile strength of 40.5 MPa; SGE–PEO/nano-clay system reached ~96% degree of polymerisation and absorbed energy of 0.8 J	Rigid bicyclic structure; bio-derived from saccharides; good coating potential	Can be brittle at high crosslink density; may require formulation control	[4,9,39,40,41]
Furans	Epoxidised furan-based monomers, including BFDE and BOMF-derived systems	*T_g_* ranges from 71 to 133 °C with tensile strength of 68–84 MPa and flexural strength of 75–96 MPa	Renewable heteroaromatic structure; useful for sustainable high-performance networks	Long-term durability data remain limited	[10,11,42,43]
Rosin acids	Epoxidised abietic acid and levopimaric acid derivatives	*T_g_* of 140 °C and modulus of 0.71–1.71 GPa	Rigid cyclic hydrocarbon skeleton may improve thermal and mechanical stability	Structural variability depending on rosin source and thermal history	[21,22]
Natural rubber	Epoxidised natural rubber systems	Flexural strength of 58 MPa and impact strength of 7.6 kJ m^−2^	Good flexibility and toughness potential	Lower stiffness and thermal stability than rigid epoxy systems	[19,20]
Lignin	Epoxidised lignin-based epoxy	Lignin/TiO_2_ hybrid epoxy system showed improved antibacterial activity, UV protection, thermal stability, and mechanical performance	Renewable aromatic backbone; high char-forming potential; multifunctional chemistry	Molecular heterogeneity and processing variability	[23,24,25,26,27]

**Table 2:** Summary of reinforcing effects of nanofillers in bio-based epoxy systems.

Nanofiller	Bio-Epoxy System	Loading	Major Enhancements	Reinforcement Mechanism	References
Graphene oxide (GO)	Tannic acid-based hyperbranched epoxy	0.1–0.5 wt.%	Tensile strength up to 161%; toughness up to 263%; elongation up to 159%	Improved dispersion and strong interfacial interaction with hyperbranched network	[36]
Reduced graphene oxide (rGO)	Epoxidised linseed oil epoxy	Not specified	Corrosion rate reduced by 5000 times; protection efficiency of 99.98%	Barrier effect restricting ingress of corrosive species	[28]
Amino-functionalised graphene oxide (AGO)	PVA/epoxidised linseed oil system	0.5 wt.%	Improved corrosion resistance and charge transfer resistance	Enhanced crosslinking and coating integrity	[29]
Graphene oxide (GO) modified with BFDE	Bis-furan-di-epoxide system	0.05–0.5 wt.%	Tensile strength up to 80%; flexural strength up to 49%; fracture toughness up to 69%	Covalent grafting and improved stress transfer	[42]
Graphene nanoplatelets (GNPs)	Vanillin-based epoxy	Up to 10 wt.%	Thermal conductivity increased by 10 times	Formation of conductive thermal pathways	[45]
Graphene nanoplatelets (GNPs)	Acrylated epoxidised jatropha oil	0.5 wt.%	Enhanced corrosion resistance	Improved barrier performance and hydrophobicity	[46]
Carbon nanotubes (CNTs)	Bio-based benzoxazine/epoxy system	0.1–0.5 wt.%	Shape recovery improved to 98%; recovery time reduced from 36 s to 16 s	Enhanced thermal transport and network reinforcement	[44]
Carbon nanofillers (CNTs, graphene, fullerene)	Tung oil-based epoxy coatings	1 wt.%	Improved hardness, thermal stability, and wear resistance	Nano-reinforcement and improved load transfer	[47,48,49]
Carbon quantum dots (CQDs)	Castor oil-based epoxy	3 wt.%	UV shielding up to 98%; improved tensile and adhesion properties	UV absorption and nanoscale reinforcement	[50]
Cellulose nanofibres (CeNFs)	Bio-based epoxy resin	0.9–1.4 wt.%	Improved tribological properties	Hydrogen bonding and fibre network reinforcement	[52]
Cellulose nanofibres (CeNFs)	Bio-based epoxy resin	Not specified	Improved tensile and fracture performance	Enhanced stress transfer through nanofibre network	[53]
Cellulose nano-whiskers (CNWs)	Terpene-based epoxy	0.5–8 wt.%	Modulus increased from 295 to 800 MPa; tensile strength doubled	Strong reinforcing effect of nano-whiskers	[54]
Cellulose nanocrystals (CNCs)	DPA-derived bio-epoxy	10 wt.%	Storage modulus increased from 19.5 to 151.1 MPa at 160 °C	Surface-modified CNCs improved matrix interaction	[57]
Surface-modified CNCs	Eugenol-based bio-epoxy	Not specified	Improved coating toughness and adhesion	Enhanced interfacial compatibility	[59]
Nano-clay (cloisite 93A)	Sorbitol glycidyl ether epoxy	5 wt.%	Degree of polymerisation up to 96%; enhanced hardness and modulus	Layered clay structure improved crosslinking and crack resistance	[39]
Nano-clay (cloisite 20B/30A)	Castor oil/SGE epoxy blends	Not specified	Improved curing and mechanical properties	Tortuous crack path and barrier effect	[61]
Modified nano-clay	Super Sap Entropy bio-epoxy	Not specified	Flexural strength up to 23–38%; modulus up to 28–37%	Improved compatibility and load transfer	[62]
Montmorillonite (MMT)	AECO/DGEBA epoxy blends	1 wt.%	Improved fracture toughness and thermal stability	Crack deflection and restricted polymer mobility	[32,33,34]
Montmorillonite	Castor oil-based hyperbranched epoxy	1–3 wt.%	Thermal stability increased from 213 to 316 °C; tensile strength doubled	Reinforced layered structure and enhanced crosslinking	[34]
Mesoporous/spherical nano-silica	Isosorbide-based epoxy coating	25 wt.%	Corrosion inhibition efficiency ~93.75%; hydrophobicity improved	Dense barrier network and surface roughness effect	[40,41]
Silsesquioxane nanoparticles	Silicon-containing bio-epoxy	1–5 wt.%	Increased tensile modulus and tensile strength	Nanoparticle crystallisation and matrix reinforcement	[65,66]
Silica nanoparticles	Furan-based epoxy/silica hybrid	Not specified	Comparable thermal performance to petroleum-based epoxy	Uniform inorganic–organic hybrid structure	[43]
Diatomite (silica-rich filler)	BEF-EP bio-epoxy	20 wt.%	Improved thermal stability, storage modulus, and hydrophobicity	Porous silica structure and barrier effect	[67]
ZnO nanoparticles	Acrylated epoxidised jatropha oil	5 wt.%	Optimum corrosion protection	Barrier effect and surface passivation	[69]
Polyaniline fibres (PAFs)	Castor oil-based epoxy	Not specified	Corrosion rate reduced to 5.68 × 10^−3^ mpy	Conductive barrier network	[70]
Chitin nanofibres	Sorbitol polyglycidyl ether epoxy	3 wt.%	Modulus reached 2390 MPa; tensile strength reached 49.6 MPa	Strong interfacial bonding through amino functionality	[71,73]
Chitin nano-whiskers	Bio-derived bisphenol-A epoxy	2.5 wt.%	Toughness up to 457%; tensile strength up to 49%	Efficient stress transfer via whisker–matrix interaction	[73]
TiO_2_/lignin hybrid nanoparticles	Lignin-based epoxy	10 wt.%	Improved UV shielding, antibacterial activity, and thermal stability	Hybrid inorganic–organic reinforcement	[72]

**Table 3:** Approximate cost comparison of conventional and bio-based epoxy feedstocks.

Feedstock/Epoxy System	Raw Feedstock Cost (US$ kg^−1^)	Epoxidation/Conversion Energy Cost (US$ kg^−1^)	Estimated Total Processing Cost (US$ kg^−1^ resin)	Approximate Final Resin Cost (US$ kg^−1^)	Main Cost Drivers
Soybean oil (ESO)	0.8–1.5	0.03–0.10	0.4–1.4	1.5–3.0	Commodity oil availability, mild peracid epoxidation
Linseed oil (ELO)	1.2–2.0	0.05–0.12	0.7–1.7	2.0–4.0	Higher unsaturation improves epoxidation efficiency
Canola oil epoxy	1.0–1.8	0.05–0.10	0.7–1.7	2.0–3.8	Lower oxirane yield and longer reaction time
Castor oil epoxy	1.5–2.5	0.05–0.12	1.0–2.2	2.5–5.0	Hydroxyl functionality increases purification demand
Karanja oil epoxy	0.8–1.8	0.05–0.12	1.0–2.2	2.0–4.0	Limited industrial scale and variable feedstock quality
Cardanol epoxy	1.5–3.0	0.08–0.20	2.0–3.7	3.5–7.0	Phenolic modification and side-chain epoxidation
Rosin-derived epoxy	2.0–4.0	0.15–0.30	3.0–6.0	5.0–10.0	Multi-step glycidylation and purification
Lignin-derived epoxy	0.1–0.8	0.30–0.70	5.5–14.0	6.0–15.0	Depolymerisation, fractionation, solvent-intensive purification
Isosorbide epoxy	2.0–5.0	0.40–1.00	5.0–15.0	8.0–20.0	Multi-stage carbohydrate conversion and glycidylation
DGEBA epoxy	1.8–3.0	0.10–0.25	1.2–3.0	3.0–6.0	Mature continuous petrochemical processing

## Data Availability

No new data were created or analyzed in this study.
